# Continuous local antibiotic perfusion technique for surgical site infections after shoulder surgery

**DOI:** 10.1016/j.xrrt.2024.04.013

**Published:** 2024-05-10

**Authors:** Yohei Shimada, Nobuyasu Ochiai, Eiko Hashimoto, Daisuke Kajiwara, Yu Hiraoka, Kenta Inagaki, Seiji Ohtori, Hisateru Niki

**Affiliations:** a Department of Orthopedic Surgery, St. Marianna University School of Medicine, Kanagawa, Japan; b Department of Orthopedic Surgery, Graduate School of Medicine, Chiba University, Chiba, Japan; c Department of Orthopedic Surgery, Numazu City Hospital, Sizuoka, Japan

**Keywords:** Continuous local antibiotic perfusion, CLAP, Surgical site infection, Shoulder surgery, Arthroplasty, Superior capsule reconstruction

## Abstract

**Background:**

Continuous local antibiotic perfusion (CLAP) is a method for preserving tissue and function against surgical site infections (SSIs) after shoulder surgery.

**Methods:**

To describe the application of the novel CLAP technique to 10 patients with SSIs after shoulder surgery that were not controlled with repeated surgical débridement or elderly patients who are insufficient physical resilience for further surgeries.

**Results:**

CLAP, consisting of gentamicin, was performed for 2 weeks, after which the infection was well-controlled. The white blood cell count and C-reactive protein level improved rapidly within 1 week of initiating CLAP, after which the patients were switched to oral antibiotics for 3 months. None of the patients experienced any adverse events.

**Conclusion:**

CLAP for SSIs after shoulder surgery was successful in preserving implants and grafts. The SSIs were controlled with no adverse events. CLAP may be an important treatment option for SSIs after shoulder surgery.

It is well-known that surgical site infections (SSIs) are among the most serious postoperative complications after shoulder surgery. Postprosthetic infections have been reported to occur in 0.2%-3.8% of patients postoperatively.^10^ The management of postprosthetic infections includes repeated surgical débridement and prolonged antibiotic therapy, as well as removal of the prosthesis, all of which are undesirable due to poor clinical outcomes.^9^ Although there is no consensus on the ideal treatment protocol for patients with SSIs following shoulder surgery; in the recent years, continuous local antibiotic perfusion (CLAP) has been reported as a novel surgical technique to treat bone and soft tissue infections with minimal tissue resection and preservation of function.^3^^,^^5^^,^^11^ CLAP is a method that involves the continuous administration of a high local antibiotic concentration, controlling the dead space by applying negative pressure to the incision and the abscess cavity site, and reducing antibiotic side effects by draining the antibiotics through a drainage tube.^3^^,^^5^^,^^11^ There are no studies, however, involving the use of CLAP for SSIs after shoulder surgery. The purpose of this study was to evaluate 10 patients with SSIs after shoulder surgery that were not controlled with repeated surgical débridement and to describe the details of the CLAP procedure.

## Methods

CLAP was performed in 10 patients with SSIs after shoulder surgery that were not controlled with repeated surgical débridement or elderly patients who are insufficient physical resilience for further surgeries. The following general parameters were evaluated: patient age; gender; primary diagnosis; surgical procedure; duration of infection before CLAP; number of débridements performed before CLAP; bacterial species causing the infection; whether or not the implant or graft was removed; whether or not the implant or graft was replaced at the time CLAP was initiated; time to C-reactive protein (CRP) negativity (<0.3 mg/dl) peak gentamicin blood levels; adverse events; reoperations; and follow-up evaluation findings.

### Surgical procedure

**Figure 1:**
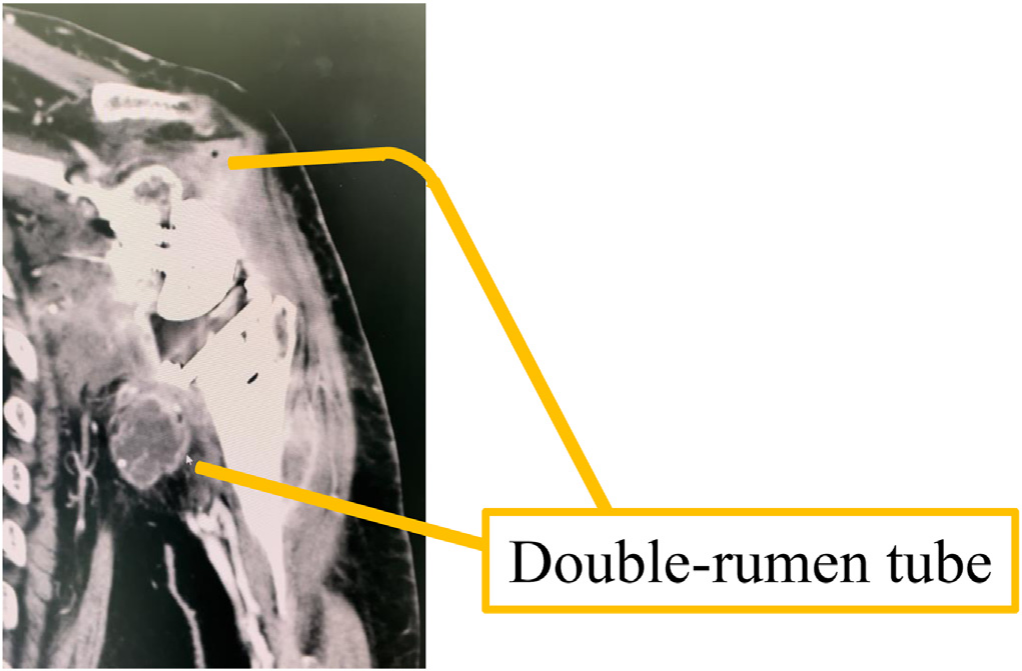
Two of the double-lumen tubes were inserted into the glenohumeral joint.

**Figure 2:**
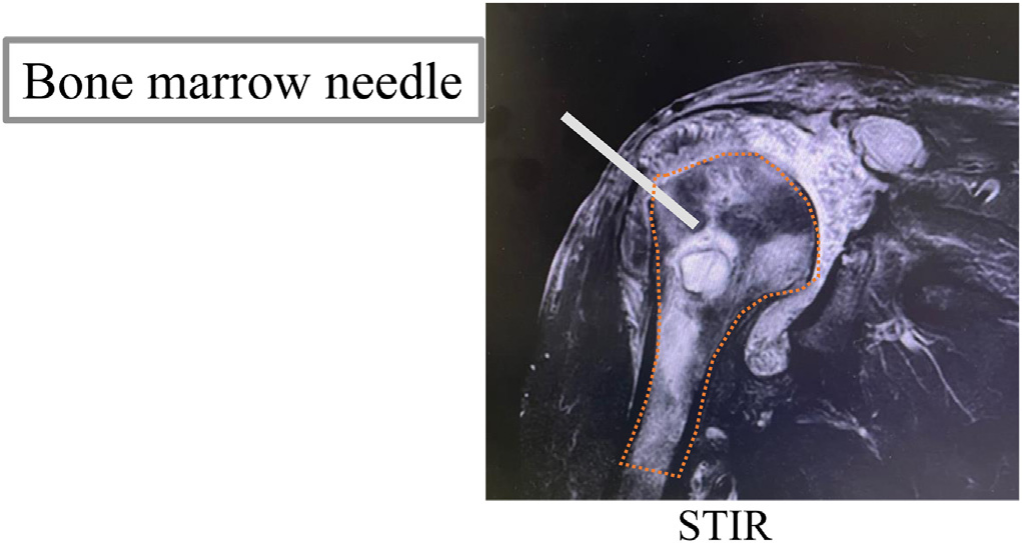
Bone marrow needle was inserted into the proximal humerus.

**Figure 3:**
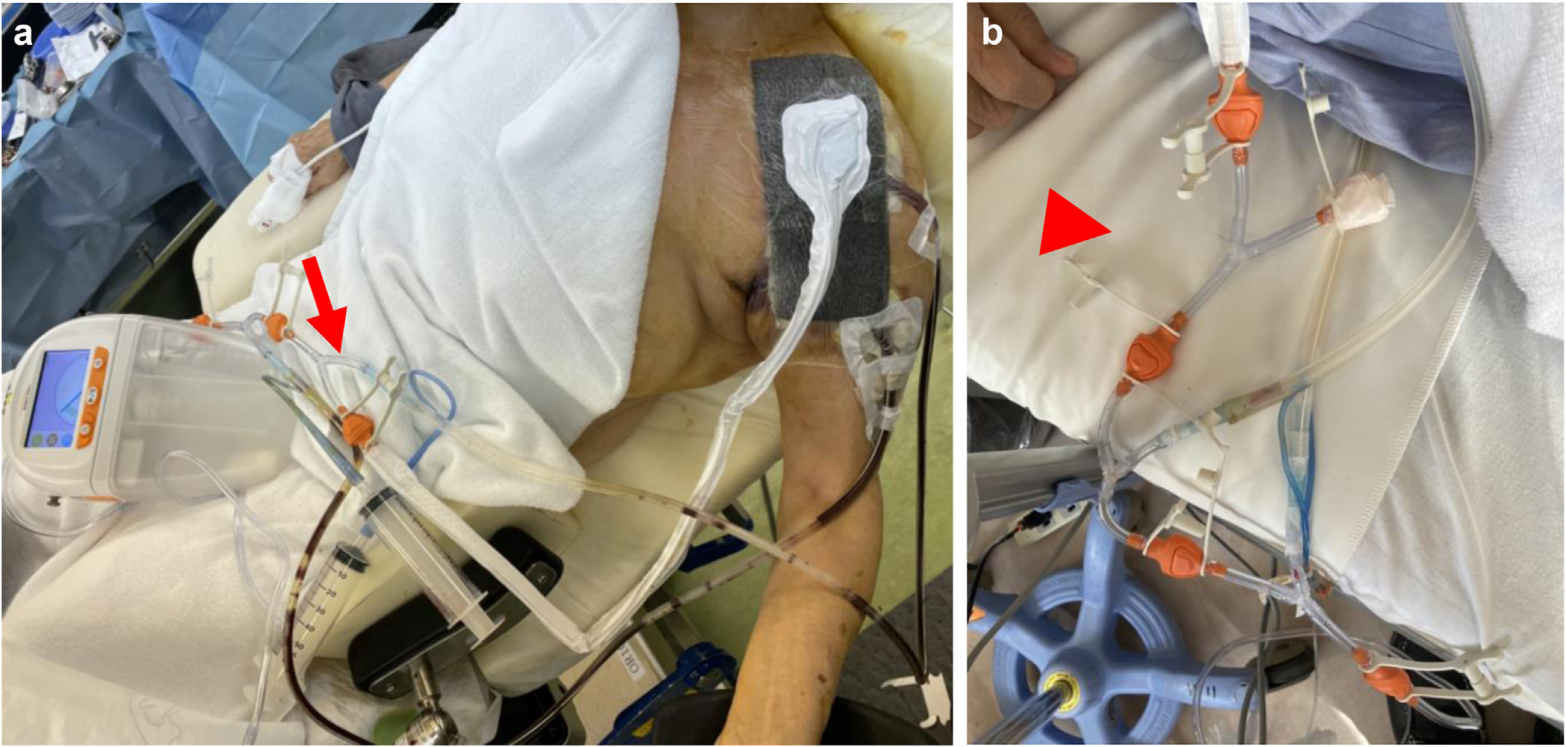
**a**, **b**. Renasys was attached to the wound (→) and a Salum-sump tube™ was connected to Renasys ().

Surgery was performed under general anesthesia in the beach chair position. The wound was exposed, washed, and débrided. In the reverse shoulder arthroplasty (RSA) cases, the easily replaceable components, such as the glenosphere, the stem liner, and the tray, were removed and partial revision surgery was performed. When stem loosening was observed, the stem was extracted and replaced using antibiotic-impregnated cement (Zimmer-Biomet, Darmstadt, Germany). The baseplate was not removed unless there was evidence of loosening due to concerns about the risk of fracture. Then, 2.16-French double-lumen tubes (Salem Sump Tube; Cardinal Health, Dublin, OH, USA) was inserted into the glenohumeral joint and subacromial space.^5^ The tips of the double-lumen tubes were placed at the most infected sites to optimize the antibiotic effect (Fig. 1). The proximal humerus was punctured with a 2.4-mm K-wire and a 3-mm outer diameter bone marrow needle (Senko Medical Instrument Manufacturing Co., Tokyo, Japan) was inserted if osteomyelitis was present with preoperative imaging determining the spread of osteomyelitis (Fig. 2). We then confirmed that the contrast medium was flowing into the bone marrow based on fluoroscopic findings (flow test). The wound was completely closed and negative pressure wound therapy (NPWT) was applied using Renasys (Smith & Nephew Medical Ltd., Kingston upon Hull, UK). Finally, a branch was created with the Renasys suction tube (Fig. 3*a*) and the suction port of the double-lumen tube was connected to the end of the branch (Fig. 3*b*).

**Figure 4:**
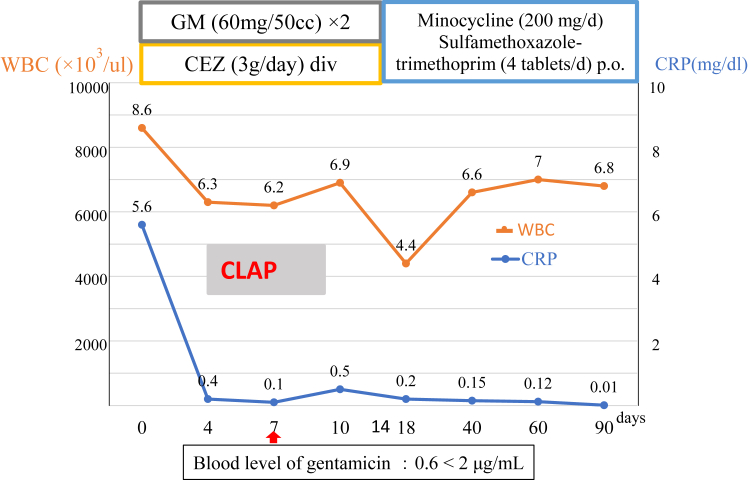
The relevant antibiotic and blood date.

### Postoperative management

Gentamicin, a concentration-dependent aminoglycoside, was the first choice for topical antibiotic administration, regardless of the susceptibility of the causative bacteria. High concentrations of gentamicin have been reported to be effective not only against methicillin-resistant Staphylococcus aureus but also against biofilm-forming staphylococci.^8^

The topical antibiotic used in CLAP was gentamicin 1A (60 mg) in 50 ml of saline administered continuously with a total of 3 continuous infusions at 2 ml/h via 2 bone marrow needles and the double-lumen tube.^5^ Wound exudate and perfused antibiotics were drained from the double-lumen tube with NPWT; the suction pressure of the NPWT was set at 80 mmHg^3^. Antibiotics were consulted with an infectious disease specialist, and intravenous administration was initially given with cephazolin (3 g/day), a standard antibiotic for SSI, and later changed according to culture sensitivity. Laboratory parameters, including the white blood cell count and CRP level, were obtained 4, 7, 10, and 14 days after CLAP was initiated. Blood gentamicin levels were measured at 4, 7, and 10 days. Patients were monitored for adverse events, including renal dysfunction and auditory neuropathy. CLAP and intravenous administration were continued for 14 days. Additionally, oral administration involves selecting 2 agents from rifampicin, sulfamethoxazole-trimethoprim, and minocycline, again based on culture sensitivity, for an empirically 3-month period.

## Results

The patient characteristics are shown in Table I. There were 6 males and 4 females with a mean age of 74 ± 17.4 years. The primary shoulder diseases included cuff tear arthropathy (n = 5), rotator cuff tears (n = 2), chronic dislocation, fracture, and pyogenic arthritis (n = 1 each). There surgical procedures performed included RSA (n = 7), superior capsule reconstruction (n = 2), and hemiarthroplasty (n = 1). The reverse shoulder prosthetic implants used in this study included the Trabecular Metal Reverse Shoulder (Zimmer Biomet, Warsaw, IN, USA) in 3 cases, the Equinoxe (Exactech, Gainesville, FL, USA) in 2 cases, the Aequalis (Stryker, Kalamazoo, MI, USA) in 1 case, and the Ascend Flex (Stryker, Kalamazoo, MI, USA) in 1 case. The mean duration of infection before CLAP was 9 ± 11.6 months and the mean number of débridements before CLAP was 2 ± 1.8. Pathogenic bacteria were methicillin-susceptible Staphylococcus aureus (n = 2), methicillin-resistant S. aureus (n = 2), *Cutibacterium acnes* (n = 1), prevotella (n = 1), and Proteus mirabilis (n = 1), and unknown (n = 3). All 10 patients were successfully treated and no adverse events were observed during CLAP. The stem was removed due to a large amount of pus and loosening following the hemiarthroplasty and in five of seven RSAs the tray, sphere, and stem were replaced during CLAP. Function was preserved in 9 of 10 patients. CRP negativity (<3 mg/dl) was obtained in approximately 7 days and peak gentamicin blood levels were all within reference values. There were no adverse events or reoperations. The mean duration of follow-up was 24 ± 11 months after the SSI healed.

**Table I:** The patient characteristics and demographic date.

Patient characteristics											
Case	1	2	3	4	5	6	7	8	9	10	Ave.
Age	90	88	73	79	87	76	46	52	75	70	74 ± 17.4
Gender; Male/Female	M	F	M	M	F	F	M	M	M	F	
Past history	None						Atopic dermatitis	None		Diabetes	
Primary shoulder disease	CTA				Chronic dislocation		RCT		Fracture	Pyogenic arthritis	
Surgical procedure	RSA						SCR		Hemiarthroplasty	Debrientment	
Duration of infection before CLAP, mo	10	8	1	12	2	1	38	5	1	12	9 ± 11.6
Débridements before CLAP, times	1	5	0	3	1	0	5	2	0	2	2 ± 1.8
Bacterial species causing the infection	None	MRSA	None	None	*P. acnes*	MSSA	MRSA	Prevotella	Proteus mirabilis	MSSA	
Removal of implant or graft	None								Stem	None	
Implant or graft replacement at the same time as CLAP surgery	Glenosphere,tray and liner						None				
Time to C-reactive protein negativity (<0.3 mg/dl), d	7	10	10	10	10	10	7	7	10	10	9 ± 1.3
Maximum gentamicin blood concentration (μg/dl)	0.6	0.7	0.5	0.3	0.6	0.3	0.9	0.6	0.9	0.4	0.6 ± 0.2
Side effects	None										
Reoperation	None										
Follow-up period, mo	24	45	10	15	16	10	24	20	48	21	24 ± 11
ROM AE (°)	110	120	130	90	100	108	60	150	108	100	108 ± 25
ER (°)	20	30	30	60	10	26	30	20	26	10	26 ± 11
ADD	L1	L1	L1	Th12	L1	L1	L1	L1	L1	L1	L1 ± 1
UCLA score	31	32	34	28	25	30	25	34	30	30	30 ± 3.3
Constant score	77	90	92	79	63	79	63	92	79	72	79 ± 12

*CLAP*, continuous local antibiotic perfusion; *CTA*, cuff tear arthropathy; *RCT*, rotator cuff tear; *RSA*, reverse shoulder arthroplasty; *SCR*, superior capsule reconstruction; *MRSA*, methicillin-resistant Staphylococcus aureus; *MSSA*, methicillin-sensitive Staphylococcus aureus; *C. acnes*, Cutibacterium acnes; *AE*, active elevation; *ER*, external rotation; *ADD*, adduction; *ROM*, range of motion; *UCLA*, University of California, Los Angeles Shoulder Score..

The final postoperative range of motion achieved was 108 ± 25^°^ of elevation, 26 ± 11^°^ of external rotation, and internal rotation at the L1 ± 1 level. The UCLA score was 30 ± 3 and the Constant score was 79 ± 12 (Table I).

## Discussion

Various treatment options for SSI after shoulder surgery have been reported, including repeated surgical débridement, prolonged antibiotic therapy, prosthesis removal, myocutaneous valves for soft tissue defects, and humeral amputation, all of which are associated with poor clinical outcomes.^4^^,^^6^^,^^7^^,^^9^ Himeno et al^3^ reported that CLAP can maintain a constant appropriate local antibiotic concentration for a long time with less invasiveness and fewer complications. Takahashi et al^11^ also reported the usefulness of CLAP in spinal SSI with preservation of the implant. SSI was difficult to control after shoulder surgery in our patients. We utilized CLAP to control the infection with minimal tissue resection and to restore function. The infections were quickly controlled without any adverse events after CLAP. In addition, the implants and grafts were successfully preserved.

Generally, osteomyelitis and implant infections are difficult to treat with antimicrobials due to the formation of bacterial biofilms, which are less penetrable by such agents. Consequently, biofilm penetration becomes a critical issue in the treatment of prosthetic joint infections.^2^ The effectiveness of common antimicrobials against bacteria is typically measured by the minimum inhibitory concentration (MIC). To exert antibacterial effects on biofilm-protected bacteria on implants; however, concentrations far exceeding the MIC are required. The effective antimicrobial concentration against biofilms is reported as the minimum biofilm inhibitory concentration and minimum biofilm eradication concentration, which are generally 100-1000 times higher than the usual concentrations.^12^ Among antimicrobials, high concentrations of gentamicin have been reported to be effective against biofilms surrounding implants.^1^ Nevertheless, the use of high concentrations of gentamicin carries risks of systemic adverse events, such as nephrotoxicity and ototoxicity^1).^ Therefore, Maruo et al^5^ have reported that local administration and retrieval of gentamicin reduces systemic complications, while still eradicating biofilms and resistant bacteria. Gentamicin was also used as the locally administered antibiotic in our patients.

The benefits and safety of CLAP have not been established; therefore, great care must be taken in determining the indications and method of implementation. Despite negative cultures, patients 3, 6, and 9 were considered for CLAP due to their high-risk profiles, including advanced age and insufficient physical resilience for further surgeries. Even in the case of a negative culture, the presence of clinical signs of infection warranted the preemptive use of CLAP. This approach reflects our cautious stance on managing potential infections in vulnerable patient groups, emphasizing the need for further research to identify which patients precisely benefit from CLAP. Also, regarding safety, although the trough level of gentamicin should be 2.0 μg/ml, the trough levels in both cases in this study were < 2.0 μg/ml; however, no adverse events were reported. Strict monitoring of the serum gentamicin level is considered important to prevent adverse events.

This report and the use of CLAP had several limitations. First, the number of patients was small and there were no controls. Although débridement and intravenous and oral antibiotics may have played an important role, we are convinced that CLAP accelerated SSI healing because of the dramatic improvement in laboratory data. Second, it is unclear when CLAP is needed. We believe that the use of CLAP for all patients with SSIs after shoulder surgery would be excessive. According to a recent report on risk factors for implant removal, CLAP should be considered for patients who have risk factors for an SSI, such as patients who do not improve after repeated surgical débridement and elderly patients who are insufficient physical resilience for further surgeries. Further cost and risk–benefit analyses are needed.

Third, the appropriate duration of CLAP, intravenous antibiotic administration and the oral administration period remains unclear. Long-term CLAP is not recommended because CLAP delivery is very uncomfortable. Considering the improvements in patient laboratory parameters in this series, 2 weeks of CLAP may be sufficient for an SSI after shoulder surgery. Because the severity of SSI varies widely among patients, we should be flexible in our approach to each patient.

## Conclusion

CLAP for SSIs after shoulder surgery was successful in preserving implants and grafts with minimal tissue resection. The infections were controlled without any adverse events. Therefore, CLAP is an effective treatment for SSIs after shoulder surgery.

## Disclaimers:

Funding: No funding was disclosed by the authors.

Conflicts of interest: The authors, their immediate families, and any research foundations with which they are affiliated have not received any financial payments or other benefits from any commercial entity related to the subject of this article.

## Appendix A Supplementary data

Supplemental section
