# Supplementary material for: Continuous local antibiotic perfusion technique for surgical site infections after shoulder surgery
Source: JSES Rev Rep Tech. 2024 May 10;4(3):419–23. doi: 10.1016/j.xrrt.2024.04.013 (PMC11329016; doi:10.1016/j.xrrt.2024.04.013)
Supplement: Supplemental section [file mmc1.docx]

**Supplemental section (Patient 1 in Table 1)**

A 90-year-old man underwent an RSA for a cuff tear arthropathy (CTA) at another hospital. Two months after surgery, a SSI was diagnosed and the patient underwent debridement at the other hospital. The SSI was not controlled and the patient was referred to our hospital. On admission to our hospital a large amount of pus was present in the wound, and the WBC count and CRP level were elevated (8600/mm^3^ [segmented neutrophils, 76%] and 5.57 mg/dl, respectively). Pus specimen cultures were negative. A contrast-enhanced CT showed deep abscess formation. We observed adherence of the abscess around the sphere, baseplate, and tray intraoperatively. The surgery consisted of washing, debridement, replacement of the sphere, tray, and stem with antibiotic-containing cement, and CLAP. Gentamicin was administered continuously via a Salem sump tube™ and drainage via a Salem sump Tube™ and Renasys. Cefazolin (3 g/day) was administered systemically. Figure 4 includes the relevant antibiotic and blood data. The laboratory parameters, including the WBC count and CRP level, improved dramatically 1 week after starting CLAP and CLAP was continued for 2 weeks. The serum gentamicin levels were measured 7 days after administration and were optimal (0.8 μg/mL). After CLAP, antibiotic therapy was empirically switched to oral minocycline (200 mg/d) and sulfamethoxazole-trimethoprim (4 tablets/d) for a duration of 3 months following CLAP.

There were no adverse events, including auditory neuropathy or renal dysfunction, and no infection recurrence during follow-up of 26 months duration after SSI healing.

The range of motion included 110^o^ of elevation, 20^o^ of external rotation, and internal rotation at the L1 level. The UCLA score was 31 and the Constant score was 77 with implant preservation.

Figure 4. The relevant antibiotic and blood data in patient 1.
